# Evaluation of reference genes for reverse transcription quantitative PCR analyses of fish-pathogenic *Francisella* strains exposed to different growth conditions

**DOI:** 10.1186/1756-0500-6-76

**Published:** 2013-03-02

**Authors:** Espen Brudal, Hanne Cecilie Winther-Larsen, Duncan John Colquhoun, Samuel Duodu

**Affiliations:** 1 Section for Microbiology, Immunology and Parasitology, Department of Food Safety and Infection Biology, Norwegian School of Veterinary Science, PO Box 8146 Dep, Oslo 0033, Norway; 2 Laboratory for Microbial Dynamics (LaMDa), University of Oslo, PO Box 1068, Blindern, Oslo 0316, Norway; 3 School of Pharmacy, University of Oslo, PO Box 1068, Blindern, Oslo 0316, Norway; 4 Section for Bacteriology, Norwegian Veterinary Institute, PO Box 750, 0106, Sentrum, Oslo, Norway

**Keywords:** Gene expression, RT-qPCR, *Francisella noatunensis*, Fish, Francisellosis

## Abstract

**Background:**

Reverse transcription quantitative PCR has become a powerful technique to monitor mRNA transcription in response to different environmental conditions in many bacterial species. However, correct evaluation of data requires accurate and reliable use of reference genes whose transcription does not change during the course of the experiment. In the present study exposure to different growth conditions was used to validate the transcription stability of eight reference gene candidates in three strains from two subspecies of *Francisella noatunensis*, a pathogen causing disease in both warm and cold water fish species.

**Results:**

Relative transcription levels for genes encoding DNA gyrase (*gyrA*), RNA polymerase beta subunit (*rpoB*), DNA polymerase I (*polA*), cell division protein (*ftsZ*), outer membrane protein (*fopA*), riboflavin biosynthesis protein (*ribC*), 16S ribosomal RNA (*16S rRNA*) and DNA helicases (*uvrD*) were quantified under exponential, stationary and iron-restricted growth conditions. The suitability of selected reference genes for reliable interpretation of gene expression data was tested using the virulence-associated intracellular growth locus subunit C (*iglC*) gene.

**Conclusion:**

Although the transcription stability of the reference genes was slightly different in the three strains studied, *fopA*, *ftsZ* and *polA* proved to be the most stable and suitable for normalization of gene transcription in *Francisella noatunensis* ssp.

## Background

Systemic infection caused by the Gram-negative bacterium *Francisella noatunensis* remains a serious threat to Atlantic cod *Gadhus morhua* L. farming in Norway. Similar diseases associated with different strains of *F. noatunensis* have been reported in both fresh- and seawater farmed fish in Taiwan and Japan [1] Chile [2], America [3], and Great Britain [4]. Large knowledge gaps exist, however, in relation to pathogenesis and mechanisms of disease development. Recent genome analysis has revealed the presence of several virulence determinant loci in fish pathogenic *Francisella* spp., which share a high degree of sequence identity with the human pathogen *F. tularensis*[5]. Some of the most interesting genes are localized on the 33-kb *Francisella* Pathogenicity Island (FPI) [6,7], including genes of the intracellular growth locus operon *iglABCD*. The *iglC* gene has also been shown to be important for virulence in *F. noatunensis* ssp. *orientalis*[8,9].

One way of investigating disease pathogenesis at the molecular level is by reverse transcription quantitative PCR (RT-qPCR) analysis of gene transcription at different stages of disease development. However, use of RT-qPCR for gene transcription studies has its pitfalls. The method has an intrinsic requirement for normalization of target gene transcription levels against that of reference genes to ensure reliable data interpretation, as exemplified by Dedha et al. [10], and Guitierrez et al. [11]. The use of a minimum of three validated reference genes has been suggested [12]. Similarly, the importance of standardization of RNA extraction techniques, evaluation of RNA quality and the use of reference genes has been emphasized in several publications [13-15].

In this study, the transcriptional stability of eight reference genes in *Francisella noatunensis* ssp. was investigated under three different environmental conditions using the excel-based software geNorm [12]. To accurately quantify changes in transcription levels of specific mRNA targets, the *iglC* gene was chosen and normalized against the selected reference genes subjected to the same growth conditions using the established protocol.

## Methods

### Bacterial strains and growth conditions

The three isolates used in this study represent different fish pathogenic *F. noatunensis* strains. *F. noatunensis* spp. *noatunensis* (NCIMB14265^T^) was isolated from diseased Atlantic cod *Gadus morhua* L. in Norway [16]. *F. noatunensis* ssp. *noatunensis* PQ 1106 was isolated from diseased Atlantic salmon *Salmo salar* L. in Chile [2]. *F. noatunensis* ssp. *orientalis* DSM21254^T^ was isolated from three-line grunt, *Parapristipoma trilinineatum* L. in Japan [1].

The strains were stored at -80°C in growth medium containing 20% glycerol. Prior to experimentation cultures were maintained on Eugon Chocolate Ferric Agar (ECFA) plates and incubated at 20 – 22°C. ECFA consists of 30.4 g/l BD Bacto™ Eugon Broth (Difco Laboratories) 15 g/l Microbiology Agar (Merck), 5% bovine blood (Håtunlab AB) and 2 mM FeCl_3_ (Sigma-Aldrich). BD Bacto™ Eugon Broth supplemented with 2 mM FeCl_3_ (EBF) was used for liquid cultures.

The experimental conditions tested included early exponential growth phase, stationary growth phase and an iron-depleted environment. For the growth phase studies, 10 ml EBF was inoculated with colony material from ECFA plates and incubated at 20 – 22°C with gentle shaking (150 rpm). Two parallel cultures were made for each strain on subsequent days. Optical density (OD) at 600 nm was measured with a Genesys 20 spectrophotometer (Thermo Scientific). For the iron depletion studies, the strains were inoculated into 10 ml EBF and incubated for 3 days at 22°C with shaking. The bacteria were subsequently pelleted by centrifugation (4000 g, 5 min), resuspended in fresh Eugon Broth without supplemented FeCl_3_ (EB) and grown for 24 hrs. Thereafter, the cultures were washed twice with PBS and once with EB supplemented with 2, 2^′^-dipyridyl (DP; Sigma-Aldrich) at a final concentration of 100 μM (EB/DP), to ensure removal of iron carried over from the previous medium. Washed bacteria were then used to inoculate cultures of EBF or EB/DP. The cultures were grown with shaking at 22°C for 24 h, after which the cells were immediately stabilized by adding 2 volumes of RNAProtect Bacteria reagent (QIAGEN).

### Primer design, PCR efficiency

Potential reference genes were chosen based on a literature review of reference genes used in RT-qPCR for *Francisella* spp. (see Additional file 1), in addition to other commonly used bacterial reference genes. Primers were designed to target eight potential reference genes and the putative virulence gene *iglC* (see Table 1). The examined reference genes were *uvrD* (helicase, separates two annealed nucleic acid strands during DNA replication, transcription, translation, recombination, DNA repair and ribosome biogenesis) *rpoB* (β subunit of RNA polymerase), *gyrA* (DNA topoisomerase II), *polA* (DNA polymerase I), *fopA* (*Francisella* outer membrane protein A), *ftsZ* (encoding a prokaryotic cytoskeletal protein important for cell division), *ribC* (riboflavin synthetase) and *16S rRNA* (small ribosomal RNA subunit). Primer efficiencies were determined using 10-fold dilution series of cDNA and genomic DNA as template for qPCR reactions.

**Table 1:** Primers used for RT-qPCR in the present study

				**Primer efficiency**			
**Target gene**	**Forward primer (5^′^- 3^′^)**	**Reverse primer (5^′^- 3^′^)**	**Amplicon size**	***Fn*. ssp. *noatunensis***	***Fn*. ssp. *orientalis***	**Gene ID**	**Position**
*uvrD*	ACTATTTGTCGCGGGTCCTT	TCAAAGAAACGAAAACCTCCA	82 bp	2.050	1.959	12951493	596634-596715
*rpoB*	GTGGTAAAGCGCAATTTGGT	CAGCACCATATGCTTGTAACG	72 bp	1.986	1.988	12951517	620011-620082
*gyrA*	CGAGCTTTACGAGCTGCTTC	TCTTTTAGAGAACCCTAAAGAGGCT	87 bp	1.982	2.000	12952071	1187616-1187702
*polA*	AGCTGGAACTGGTCGTAATCA	ATCAGCATCTTCAGCAGCATA	82 bp	1.959	1.950	12951484	584274-584355
*fopA*	TACTGGTGCATGGGATGTTG	TCTTGGAGCCATTGTCTGAA	100 bp	1.902	1.938	12952182	1297252-1297351
*ftsZ*	TACCATACTCAGCGGCTTTC	GCGCCTGTAGTTGCTGAAGT	112 bp	1.986	1.997	12952792	136099-136210
*ribC*	ATCTCAACTAGCCACGCTCC	CGGTGGACACATGGTACAAG	87 bp	1.946	1.950	12952738	84136-84222
*16S rRNA*	AACGACTGTTAATACCGCATAATATCTG [17]	CCTTACCCTACCAACTAGCTAATCCA [17]	101 bp	1.954	1.954	12951375	463870-463970
*iglC*	TAGGCGTATAACACTGGCTGC	TGCTATAGAAGGCGGAGAGG	70 bp	2.006	1.905	12951826	930145-930214

The complete genome of *F. noatunensis* ssp. *orientalis* strain Toba 04 (Accession number NC_017909), was used as a reference to assign the Gene ID.

### RNA isolation and reverse transcription

Bacterial cells were stabilized with RNAProtect Bacteria Reagent (QIAGEN) and total RNA extracted from 500 μl early exponential growth phase cultures and 250 μl stationary growth phase cultures using RNeasy Mini Kit (QIAGEN) according to the manufacturer’s instructions. A 15 minute on-column DNase digestion with RNase-Free DNase (QIAGEN) was performed to ensure removal of contaminating genomic DNA, as suggested by the manufacturer. For each strain, three RNA extractions were performed from exponential and stationary growth phases, while two extractions were performed for the iron-depletion experiment. RNA concentration and purity, determined by 260/280 and 260/230 ratios, were measured with a Nanodrop ND-1000 spectrophotometer (Nanodrop Technologies Inc.). RNA integrity was assessed by gel electrophoresis as described by BioRad Technical Note 5396 [18] using 500 ng of the extracted RNA and evaluation of the ratio between the bands corresponding to 16S and 23S ribosomal RNA. Reverse transcription of 1 μg extracted RNA in 20 μl reactions was performed with QuantiTect Reverse Transcription kit (QIAGEN) using random primers according to the manufacturer’s instructions. A control with omitted reverse transcriptase was performed for each extraction to check for the presence of contaminating genomic DNA. After reverse transcription, the samples were diluted 1:10 in DEPC treated H_2_O and used as templates for qPCR.

### Reference gene validation

Quantitative PCR carried out with the Stratagene Mx3005 thermal cycler (Stratagene, La Jolla, San Diego, CA) was performed in a 25 μl reaction volume containing 2 μl of the appropriate cDNA or genomic DNA, 12.5 μl 2 × High power SYBR green PCR Master Mix (Applied Biosystems) and 300 nM concentration of the appropriate forward and reverse primers (Invitrogen). The thermal cycling conditions for the PCR were as follows: 1 cycle at 95°C for 10 min, 45 cycles of amplification at 95°C for 15 s and annealing at 60°C for 1 min. The data were collected during each elongation step. Melting-curve analysis consisting of 1 cycle at 95°C for 30 s, 55°C for 30 s and 95°C for 30 s was also performed after SYBR green I PCR to check the specificity of the amplification products. Negative (DEPC treated H_2_O) and no-reverse transcriptase controls were included in each run. All samples and controls were analyzed in triplicate.

The data was analyzed using the excel-based software geNorm [12], which compares the relative expression of reference genes in a pair-wise manner, and awards an M-value (expression stability) to each gene. The lowest M-value corresponds to the most stable reference gene. The worst scoring reference gene was then excluded from the analysis, and the process repeated in a step-wise manner until only the two best reference genes remained. The reference genes were ranked 1-8, with the most stable reference gene given the lowest value. The overall stability of each reference gene was determined by the sum of the ranking values from all four datasets combined. For calculation of the number of reference genes needed for reliable quantification of a target gene, the recommended cut-off for the pair-wise variation value V of 0.15 was used.

### Normalization of *iglC* transcription

The relative transcription of the potential virulence gene *iglC* was investigated using the reference genes recommended by geNorm. For NCIMB14265^T^ and DSM21254^T^, the five most stably transcribed reference genes were used, while for PQ 1106, the four most stably expressed reference genes were used. The relative transcription of *iglC* was determined in all samples by normalization against a normalization factor calculated by geNorm (based upon the geometric mean of the selected reference genes). The normalized values were divided by the arithmetic mean of the normalized log-phase expression values of *iglC* of each strain for presentation purposes. The statistical analysis of the normalized *iglC* transcription data was performed using JMP 8.0.2. (SAS Institute Inc.). The difference in *iglC* transcription levels between different growth phases, and the effect of an iron-depleted medium, were compared and determined as statistically significant if *p*-value <0.05 by Student’s t-test assuming unequal variance.

## Results

### Primer specificity and efficiency

Melting curve analysis revealed good specificity for the target genes with single peaks obtained for all primer sets. The primer efficiencies and amplicon size are shown in Table 1.

### Transcription and stability of reference genes

**Figure 1:**
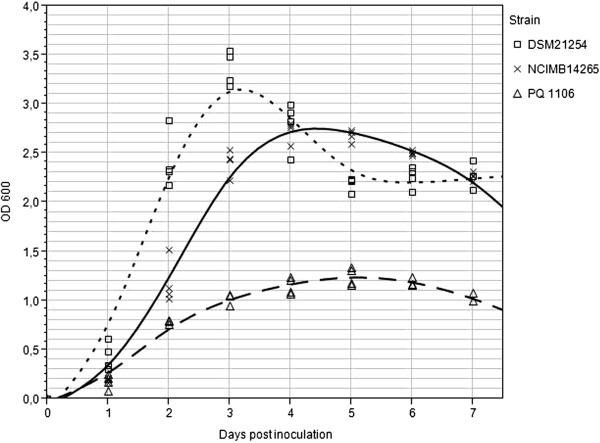
**Growth curves for the strains used in the study.** NCIMB14265 = *F. noatunensis* ssp. *noatunensis* strain NCIMB14265^T^. PQ 1106 = *F. noatunensis* ssp. *noatunensis* strain PQ 1106. DSM21254 = *F. noatunensis* ssp. *orientalis* strain DSM21254^T^. The growth curve for NCIMB14265^T^ corresponds well to previously published data [19]. The two *F. noatunensis* ssp. *noatunensis* strains reach stationary growth phase at the same time point, after approximately 5 days, although PQ 1106 grows to lower OD_600nm_ compared to NCIMB14265^T^. DSM21254^T^ reaches a higher OD, and stationary growth phase after 3 days.

**Figure 2:**
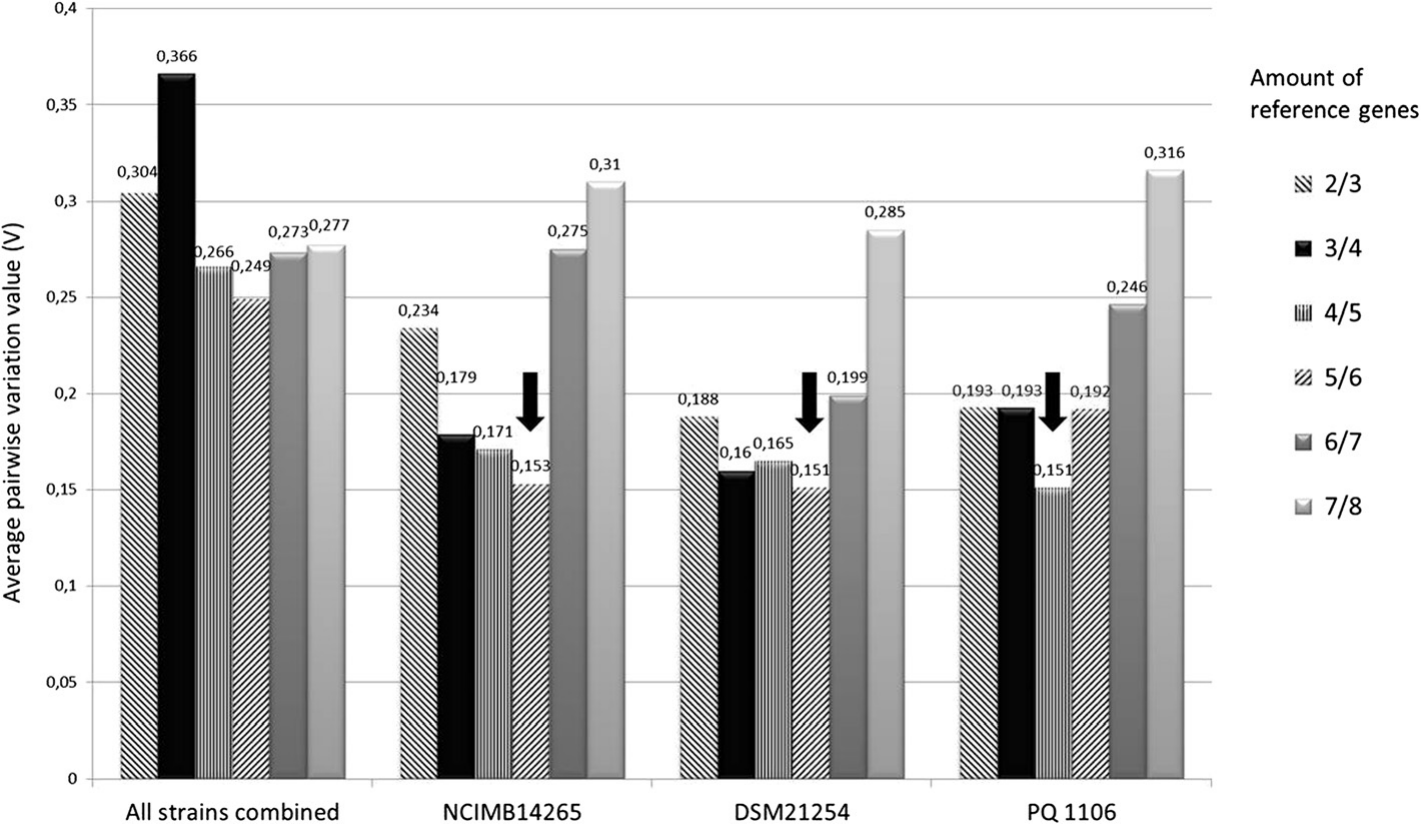
**Average pairwise variation value V.** NCIMB14265 = *F. noatunensis* ssp. *noatunensis* strain NCIMB14265^T^. PQ 1106 = *F. noatunensis* ssp. *noatunensis* strain PQ 1106. DSM21254 = *F. noatunensis* ssp. *orientalis* strain DSM21254^T^. Arrows indicate the number of reference genes needed for accurate normalization of a target gene for each strain.

To identify stably expressed reference genes in *F. noatunensis* ssp., eight candidate reference genes were initially analyzed. The bacterial cells were harvested in early exponential (OD_600nm_ 0.3-0.5) growth phase and upon entry into stationary phase at 3 and 5 days post-inoculation for ssp. *orientalis* and ssp*. noatunensis* strains, respectively (see Figure 1). The purity of extracted total RNA used for reverse transcription was good, with 260/280 ratios above 2.1, and 260/230 ratios above 2.2 in all samples. No RNA degradation was observed as evaluated by gel electrophoresis. The data from the three strains combined suggested *polA*, *ribC* and *ftsZ* as the most stably transcribed reference genes across all strains, as indicated by the lowest M-values (Table 2). The pooled data, however, could not be used for analysis due to substantial pairwise variation (Figure 2). On individual analysis, we identified stably expressed reference genes for each strain. For *F. noatunensis* ssp. *noatunensis* NCIMB14265^T^, *fopA*, *ftsZ* and *ribC* were the three most stably transcribed genes, while *ftsZ*, *polA* and *fopA*; and *ftsZ*, *gyrA* and *polA* were the three most stably transcribed genes in *F. noatunensis* ssp. *noatunensis* PQ 1106 and *F. noatunensis* ssp. *orientalis* DSM21254^T^, respectively. Regardless of strain studied, *16S rRNA* and *rpoB* consistently scored worst, and therefore constitute poor reference genes for gene transcription studies in *Francisella noatunensis* ssp. The optimal number of reference genes for each strain was determined based upon the average pairwise variation value V calculated by geNorm (Figure 2). For NCIMB14265^T^ and DSM21254^T^, the optimal number of reference genes is five, while for PQ 1106 the optimal number is four.

**Table 2:** Expression stability index (M-value) and ranking of the candidate reference genes

				**M-value**				
**Strain**	***ftsz***	***polA***	***fopA***	***ribC***	***gyrA***	***uvrD***	***16S rRNA***	***rpoB***
**NCIMB14265**^**T**^	**0.531**	**0.818**	**0.531**	**0.751**	**0.949**	**1.050**	**2.004**	**2.563**
**DSM21254**^**T**^	**0.555**	**0.692**	**0.716**	**1.009**	**0.555**	**0.892**	**1.496**	**2.343**
**PQ 1106**	**0.603**	**0.603**	**0.653**	**0.842**	**0.880**	**1.241**	**2.570**	**1.781**
**All strains combined**	**0.979**	**0.790**	**1.535**	**0.790**	**1.498**	**1.675**	**2.411**	**2.126**
**Ranking order**	**1**	**2**	**3**	**4**	**5**	**6**	**7**	**8**

The lowest M-value corresponds to the most stable gene.

### Effect of growth phase and culture conditions on *iglC* transcription

**Figure 3:**
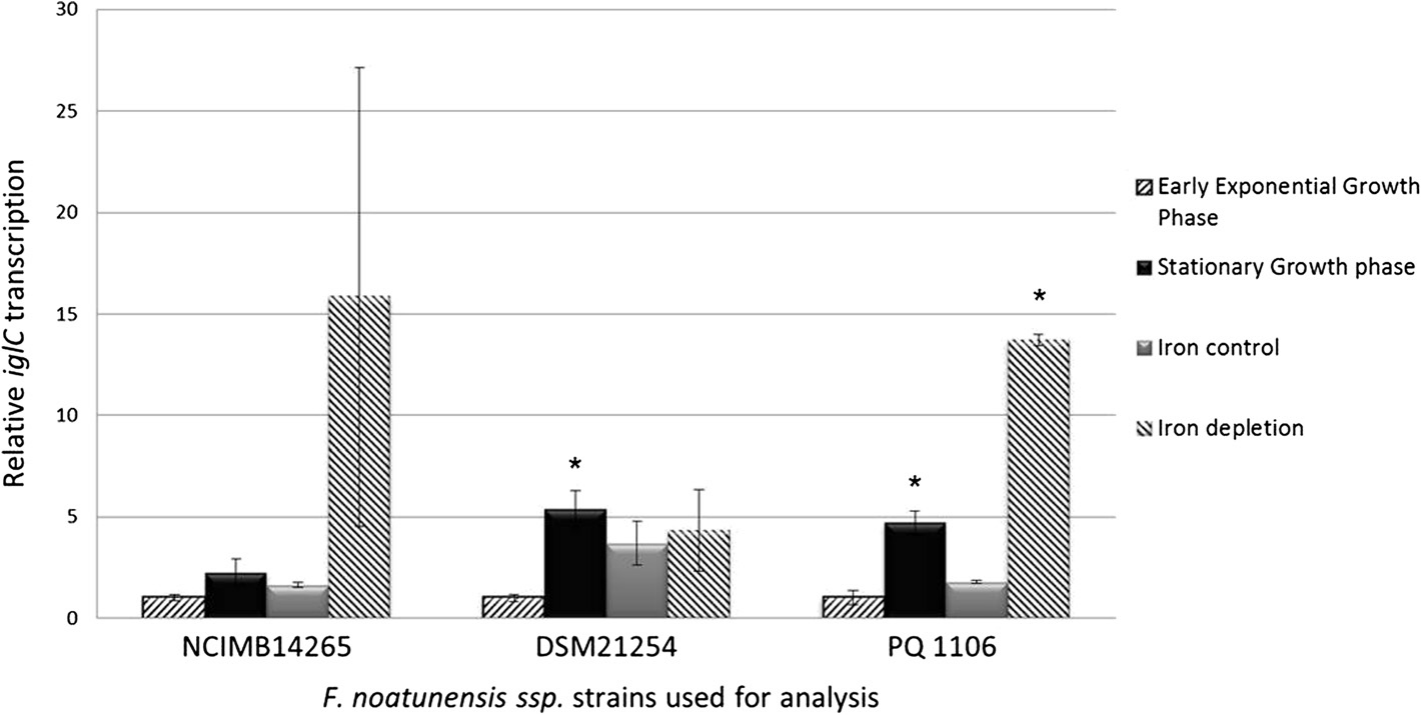
**Statistical analysis of differential transcription of *iglC *under different growth conditions.** Stars denote statistically significant differences with p-value <0.05. Bars indicate standard error of the mean. NCIMB14265 = *F. noatunensis* ssp. *noatunensis* strain NCIMB14265^T^. PQ 1106 = *F. noatunensis* ssp. *noatunensis* strain PQ 1106. DSM21254 = *F. noatunensis* ssp. *orientalis* strain DSM21254^T^.

The selected reference genes were used to assess the possible differential expression of *iglC*, which is known to be regulated by environmental cues [20,21]. All three strains displayed increased transcription of *iglC* in stationary compared to exponential growth phase, although for strain NCIMB14265^T^ the increase was not statistically significant (see Figure 3). Transcription increases of 3.69 fold and 4.32 fold were recorded for *iglC*_PQ 1106_ and *iglC*_DSM21254_^T^, respectively. In an iron-depleted medium, PQ 1106 displayed a significant 6.72 fold increase in *iglC* transcription and a trend towards increased transcription was identified in NCIMB14265^T^ (8.79 fold). No such increase was observed in DSM21254^T^.

## Discussion

Identification of bacterial reference genes stably transcribed in the stationary growth phase can be challenging [22]. This is probably due to the physiological changes occurring in a bacterial culture on adaption to a nutrient-depleted environment, as reviewed by Navarro Llorens et al. [23]. We did, however, identify several reference genes which are stably transcribed during exponential growth, stationary growth and in an iron-depleted environment. While transcription stability of the various reference genes varied slightly in the three strains, *ftsZ*, *polA* and *fopA* provided relatively good scores for individual strains with geNorm expression stability measures below 1.0, which has been regarded as suitable for normalization in several studies [24-26]. Subspecies differences were apparent in our dataset. The four top scoring reference genes were identical in both strains of *F. noatunensis* ssp. *noatunensis,* although they ranked differently*.* The observed differences between ssp. *noatunensis* and *orientalis* may be explained by inter-strain evolutionary distances. Although several genes may appear useful for evaluation of gene transcription in the tested *F. noatunensis* strains, *16S rRNA* and *rpoB* should be discouraged unless these genes are properly validated for each experimental condition.

On examination of *iglC* transcription in relation to bacterial growth phase, all strains show a trend toward higher *iglC* transcription in stationary compared to early exponential growth phase. The increase was, however, only statistically significant in two of the three studied strains (PQ 1106 and DSM21254^T^). The *iglC* gene has been shown to be important for intracellular growth in *F. noatunensis* ssp. *orientalis*[27]. The nutrient-depleted environment encountered in the stationary growth phase may mimic some aspects of the intra macrophage environment. Virulence associated factors, up-regulated in the stationary growth phase have also been shown in other intracellular pathogenic bacteria [28,29] to be up-regulated in infected macrophages. Our results indicate that transcription of *iglC* is increased in an environment mimicking intracellular conditions, thus suggesting a virulence-related intracellular survival role for this gene in *F. noatunensis* ssp.

The overall increase in *iglC* transcription in *F. noatunensis* ssp. *noatunensis* strains in an iron-depleted environment is consistent with the situation in *F. tularensis* ssp. *holarctica* Live Vaccine Strain [20]. Exposure to iron-limitation after reaching stationary growth phase might have prevented further induction of *iglC* transcription in DSM21254^T^. This is partially supported by data from the early logarithmic growth, where a 3.31 fold increase in *iglC* transcription was observed.

Generally, analysis of RT-qPCR gene expression data using a single reference gene is not acceptable, as inclusion of multiple reference genes results in much more accurate and reliable normalization. The geNorm calculated V values give the optimal number of reference genes to be used in an expression study [12]. Although the use of three reference genes is a valid normalization strategy in most cases, our results showed that four to five reference genes are required to achieve accurate normalization of gene transcription in all *F*. *noatunensis* ssp. Besides increasing the workload and cost, applying a large reference gene set could also pose constraints on limited sample availability. On normalization of *iglC* transcription with the three most stable genes across all three strains, we achieved basically the same results as normalizing with the optimal number of four or five reference genes, but with a slightly higher degree of variation (data not shown). Thus, it is always a trade-off between accuracy and practical considerations when it comes to the optimal number of reference genes to include in the analysis. For accurate quantification of small changes in gene transcription, it might be desirable to use the specified optimal number of reference genes. However, if only large differences in gene transcription are of interest, use of a smaller number of stably expressed reference genes might be justifiable.

In conclusion, the present study investigated the most reliable reference genes for normalization of gene expression data in *F*. *noatunensis* under different *in vitro* growth conditions. Although there were many potential suitable references genes, *ftsZ*, *polA* and *fopA* were the best, while *16S rRNA* and *rpoB* proved to be the least stable under all tested conditions. These data emphasize the need for proper validation of candidate reference genes in any experimental expression study. Extrapolation of results from one strain to another must therefore be done with extreme caution. It also provides baseline data on selection of reference genes for many future studies investigating expression of virulence in pathogenic *Francisella* strains.

### Availability of supporting data

The dataset supporting the results of this article is included within the article (and its additional file).

## Competing interests

We declare no competing interests.

## Authors’ contributions

BE performed the experiments, analyzed and interpreted the data and wrote the manuscript. WLHC and CDJ were involved in the design, interpretation of the data and writing of the manuscript. DS designed and performed some of the experiments, was involved in the interpretation of the data and writing of the manuscript. All authors read and approved the final manuscript.

## Supplementary Material

Additional file 1 **List of reference genes used in publications for RT-qPCR gene expression analyses in *Francisella*** spp. [30-43]. Click here for file
